# Parsing human rights, promoting health equity: reflections on Colombia’s response to Venezuelan migration

**DOI:** 10.1093/medlaw/fwac053

**Published:** 2023-01-10

**Authors:** Stefano Angeleri, Thérèse Murphy

**Affiliations:** Health & Human Rights Unit, School of Law, Queen’s University, Belfast, UK; Marie Skłodowska-Curie Visiting Scholar, Universidad del Rosario, Bogotá, Colombia; Health & Human Rights Unit, School of Law, Queen’s University, Belfast, UK; Raoul Wallenberg Visiting Chair, Lund University, Lund, Sweden

**Keywords:** Health equity, Health inequalities, International human rights law, Irregular migrants, Right to health, Temporary Protection Statute for Venezuelan Migrants

## Abstract

Over the last 7 years, a multidimensional crisis in Venezuela has resulted in massive emigration. Over 7 million have fled the country, with more than 2.4 million seeking to settle in Colombia. Of these, as of 2021, more than 1 million were undocumented, but the situation has started to change with the implementation of an ambitious migrant regularisation scheme. Regularisation promises access to comprehensive healthcare, full educational opportunities and the formal labour market. Securing these social determinants of health is critical because social inequalities produce health inequalities—that is, systematic health differences that are preventable and thus unjust. Social medicine, social epidemiology and international human rights law agree on this, yet law-focused studies of health equity initiatives remain rare. Aiming to reverse this, we examine Colombia’s response to Venezuelan migration, including its recent migrant regularisation initiative, which was introduced in part to comply with the country’s obligations under international human rights law. The examination foregrounds what we are calling ‘legal literacy’, testing the hypothesis that advancing health equity involves asking more and better questions about international human rights law.

## I. INTRODUCTION

This article starts from a strong but straightforward hypothesis. It argues that all those concerned with health inequalities—the social injustice that is ‘killing people on a grand scale’^1^—need to ask more and better questions about international human rights law. Doing this would bolster what we call ‘legal literacy’, which would bring multiple benefits. It would deepen and broaden attention to the social determinants of health within international human rights law,^2^ which makes explicit reference to these determinants as part of the right to health.^3^ It would counter claims that human rights and equality are separate or even antithetical.^4^ It would challenge the argument that implicitly including the right to health in policymaking is sufficient; it would instead prioritise explicit use of law-based human rights language, including the rights and obligations arising from the right to health.^5^ It would forge fuller descriptions of law’s place in relations between different actors. Where these relations are unjust, it would create opportunities to reshape them, not least because when relationships involve rights and obligations, they also involve accountability in different forms—including but not limited to strategic litigation.^6^ Above all, at a time when the human rights movement is awash with commentaries on its flaws, it would encourage more rounded assessments of international human rights law’s capacity to contribute to reducing health inequities—assessments of what international human rights law can and, equally, what it cannot do. The aim is not to make strong causal claims about international human rights law’s role; success and failure are never attributable to law alone. Instead, the aim is encourage greater curiosity concerning international human rights law’s role: how is international human rights law being mobilised, by whom, with what hurdles and with what effects on health inequalities?

To make this argument, the article takes two steps. First, it sketches some standard ways of thinking and talking that flatten engagement with law and international human rights law in particular. Second, aiming to limit this, the article uses a real-world example to test the hypothesis that better legal literacy would boost efforts to advance health equity. The example is provided by Colombia’s responses to Venezuelan migration, including the Temporary Protection Statute for Venezuelan Migrants (TPSV), the country’s newest migrant status regularisation scheme. This scheme addresses a marginalised and vulnerable population of more than 2 million people—a group with state-designated illegal or precarious migratory status. It promises them regularisation and thus access to a range of the social determinants of health; it promises, in other words, to reduce the inequality gap between nationals and non-nationals.

We chose this example with care. On the one hand, Colombia is a place where international human rights law has a handhold.^7^ On the other hand, its migrant regularisation initiative addresses a hard case. Around the world, significant inflows of poor non-nationals, particularly undocumented migrants, are generally seen as a threat. Nationalist and other discourses dwell on the costs, including the different identities, of both migrants in general and undocumented migrants in particular.^8^ And as Catherine Dauvergne has pointed out, the ‘illegalisation’ of migration is rampant, and migration law and its enforcement are widely seen as ‘the last bastion of sovereignty’.^9^ Law, in other words, is widely positioned as a way of stripping rights, a way of promoting and securing the ‘rightlessness’ of migrants and institutionalising inequality.

Faced with this backdrop, we were intrigued: Was Colombia swimming against the tide, with a migrant regularisation scheme and other legal and operational initiatives that take human rights-based action on health and social inequalities? This article explores that question, drawing on a review of scholarly and policy literature, as well as empirical work in Colombia.^10^ The latter, focused on key stakeholders, had two elements. First, direct, and participant observation with the Jesuit Refugee Service (JRC) and the International Organization for Migration (IOM) in the capital, Bogotá, and two departments, Cundinamarca and Norte de Santander.^11^ These observations occurred during occasional visits in the period February−May 2022. They were designed to better understand field activities and the relationships between different actors, and they included a series of informal interviews with stakeholders. Second, semi-structured interviews with administrative, managerial and clinical staff across 18 public institutions and humanitarian and community organisations, conducted between May and August 2022.^12^

The structure of the article is as follows. Section II sketches our legal literacy hypothesis. Section III adds relevant background on Colombia, which sets the scene for Section IV in which we assess the country’s responses to the wave of migrants fleeing Venezuela. The Conclusion returns to the literacy hypothesis, offering some final observations.

## II. LEGAL LITERACY

We begin with a question: Why is there still so little engagement between international human rights law and social medicine/social epidemiology when these fields share a commitment to advancing health and health equity?^13^ Part of the answer could be that, even amidst widespread constitutionalisation and other significant strides, economic and social rights continue to be viewed by many as policy objectives rather than as law.^14^ Another part of the answer may lie in a broader phenomenon: the flattening of law’s capacity by familiar turns of phrase. Consider, first, the turns of phrase ‘law and justice’ and ‘law and ethics’. They seem to shrink law’s salience; they make it seem less interesting, less expansive than its counterparts. Positioned alongside justice or ethics, law appears to be about obligations and associated practical fixes. If this happens, law’s normative salience is lessened or lost, and it is valued mostly for its capacity to prohibit or compel, which in turn encourages unreasonable expectations about the ‘power of law’, and frustration and dismissal when legal rules, processes or outcomes seem contrary to justice or ethics.

International human rights law, the focus of this article, faces extra hurdles on the ‘power of law’ front.^15^ When national law is taken as the template, international human rights law’s norms seem imprecise and ambiguous, as well as easy to ignore if—as is the case with the right to health—they are subject to standards such as ‘progressive realisation’. To compound matters, international human rights law’s enforcement architecture can also seem unconvincing when compared with its domestic counterpart.

‘Human rights and equality’ is another popular but problematic turn of phrase. It suggests that non-discrimination and equality are related to, but separate from, human rights rather than integral thereto. It obscures equality and non-discrimination as cross-cutting principles and norms of international human rights law, including their status as ‘immediate obligations’ (that is, states have to take steps now, not later, to realise them).^16^ It also obscures international human rights law’s focused concern with poverty^17^ and its increasing attention to the effects of privatisation and commercialisation on human rights.^18^

References to ‘law and rights’ are also problematic. Amartya Sen was right to warn against ‘entirely law-dependent views of human rights’; as he says, ‘we need to see human rights … over a much bigger arena’.^19^ However, if law drops out of view in that bigger arena—if we stop being curious about it—we are not seeing the full picture. It is precisely this concern that led us to the hypothesis that better legal literacy would boost efforts to advance health equity. The hypothesis, to be clear, is not about lectures from lawyers on legal texts or about mobilising for more law or more lawyers. Nor is it about giving law a governing role in human rights or health equity. It is about *building curiosity about law’s capacity—*specifically, asking more and better questions about international human rights law’s capacity to advance health and health equity.

The hypothesis draws deeply on a new trend in law-focused human rights scholarship.^20^ Modelled in part on pioneering work by authors including anthropologist Sally Engle Merry and political scientist Beth Simmons,^21^ pockets of human rights law scholarship are now looking at advocacy campaigns, programmes and policies in detail and with openness about the place of international human rights law. This scholarship uses a range of methods, eg it includes both ethnographic research and Big Data.^22^ It also includes both top-down studies (examining, eg the UN human rights treaty bodies, and domestic and international courts and tribunals) and bottom-up ones (examining, eg who chooses to mobilise for what rights using law, how they mobilise and what obstacles they face, as well as who feels they have to live ‘out of sight’ of law as opposed to seeking its protection). In addition, it recognises that international human rights law’s capacity may not be ‘a question of bottom up or top down, but rather of multiple and often carefully tailored forms of interaction and reinforcement’, between domestic activism and international and domestic accountability.^23^

Influenced by this, and keen to bring its insights to the health equity field, we use the remainder of the article to examine Colombia’s recent migrant regularisation initiative, which was introduced in part to comply with obligations under international human rights law. The question we ask is: what does this initiative and, just as importantly, the activities that surround it, tell us about law’s capacity to advance health equity between nationals and non-nationals? More generally, we ask: does this case study support the legal literacy hypothesis—namely, that greater curiosity concerning law’s capacity would advance health equity?

## III. THE BACKGROUND

### A. The Venezuelan exodus and the Colombian response

Over the last 7 years, more than 7 million Venezuelans have fled their country due to a protracted economic, political, and social crisis that has had a profound impact on daily life.^24^ The latest available figures give a sense of the staggering scale of the crisis: in 2012, 25 per cent of people in Venezuela were living below the poverty line; by 2021, the figure was 94.5 per cent, with 76.6 per cent living in extreme poverty.^25^ International actors have observed violations of civil, political, and socioeconomic rights in the country, including torture and arbitrary detention.^26^ Relatedly, the public healthcare system has been collapsing under the weight of underfunding, shortages of essential drugs and medical devices, and the re-emergence of eradicated infectious diseases.^27^

Amidst the exodus, Colombia is the neighbouring country to which more than 2.4 million Venezuelans have to date fled.^28^ It has been a first choice for reasons including geographic proximity, historic migration patterns, and cultural similarities. Its openness has been crucial too, notably its pro-immigrant political discourse (which was particularly strong in the early years of the crisis), migrant legalisation programmes, weak immigration enforcement and welcoming of hundreds of international agencies and humanitarian NGOs onto its territory to help it meet migrants’ needs.

The country has generally left its borders open to people holding a Venezuelan passport. However, because these passports are costly and more generally difficult to obtain, irregular crossings, transits, and unauthorised stays have become routine across the 2,000-kilometre border between Colombia and Venezuela, which is mainly controlled by armed groups that operate as smugglers rather than the immigration authorities. In 2017, Colombia began adding a new dimension to its response: a series of targeted migrant regularisation schemes, which were developed under the executive powers of the government. The initial schemes, launched between 2017 and 2020, were mostly addressed to migrants who had *regularly entered* the territory, with successful applicants being given a two-year temporary stay permit.^29^ In 2021, in response to a steady flow of *irregular* immigration and the presence of at least 1 million undocumented or irregular migrants,^30^ the government launched a more ambitious scheme, the TPSV.^31^

The TPSV targets Venezuelan migrants and asylum seekers who fulfil certain eligibility criteria,^32^ including being irregularly present on the territory on or before 31 January 2021 and not having pending administrative or criminal proceedings. Unlike previous schemes, successful applicants are issued a 10-year temporary protection permit (TPP), which is designed to grant access to the formal labour market, full educational opportunities and comprehensive healthcare, and eventually the opportunity to apply for an R visa, a renewable residence visa that is a pathway to indefinite stay in Colombia.^33^ A TPP, in short, promises enjoyment of human rights, including economic and social rights, on the same basis as citizens. In health inequalities language, the overall scheme promises the conditions for ‘people to take control of their own lives’,^34^ thereby reducing social inequalities between Colombia’s migrant and national populations.

Efforts to get the Venezuelan migrant population registered under the TPSV are promising: almost 2.5 million people have registered using the scheme’s online platform and around 1.6 million TPPs have been issued. Both registration and processing have given priority to, or made accommodations for, groups classified as ‘vulnerable’ by international human rights law, including pregnant women, persons with disabilities, transgender people, and (certain categories of) children.^35^

The primary drivers of the TPSV were migration control and socioeconomic integration of a vast undocumented population, however human rights—including Colombia’s international human rights law obligations—were relevant too. Human rights terminology features significantly in both of the scheme’s texts: the decree of the Minister of Foreign Affairs, which set-up the scheme’s objectives and key features, and the implementing resolution, developed and issued by Colombia’s migration management agency, Migración Colombia.^36^ The decree contains 37 references to the realisation of fundamental or international human rights for migrant adults and children. These rights are presented as either the goal of the regularisation scheme or a key factor prompting its adoption. In particular, the decree notes that new migration policy instruments are necessary to guarantee the rights and social integration of irregular migrants, who are particularly exposed to the risk of being exploited and trafficked and who fall outside existing regularisation instruments.^37^ It emphasises that human rights treaties are a primary source of law in Colombia’s domestic legal order and that this new regulatory response to a mixed migration of migrants and refugees will contribute to domestic compliance with international obligations.^38^

The implementation resolution, which adds operative details about the scheme, also relies heavily on human rights arguments to contextualise the adoption of the scheme. It focuses, in particular, on the need to respect both binding and non-binding international human rights commitments on children’s rights and the right to work, both of which constitute important determinants of health.^39^

### B. The pre-TPSV legal context

We have seen that the TPSV and prior related initiatives were a response to a profound and protracted wave of migration from crisis-hit Venezuela, but to what extent was Colombia’s broader legal culture a prompt for them? In particular, does it appear that the country’s international human rights law obligations played a role?

In Colombia, ratified human rights treaties are a primary source of law: they constitute a standard of interpretation for the rights found in the country’s constitution and they have broad normative effects where there is no directly applicable provision in the constitutional order.^40^ They direct law-making and adjudication and contribute to making non-compliant subordinate regulations null and void.^41^

Further, in line with a broader regional trend,^42^ socio-economic rights are central entitlements in the Colombian constitutional system, and the Colombian Constitutional Court (CCC) has declared that the right to health is a directly justiciable fundamental right.^43^ In addition, the reduction of health inequalities across social groups is a clear principle of Colombia’s 2015 Health Act,^44^ and both this law and the just-cited CCC judgment that contributed to its introduction,^45^ were deeply influenced by international human rights law—in particular by the approach taken in General Comment no. 14, the interpretive guidance on the right to health issued by UN Committee on Economic, Social and Cultural Rights (CESCR). Notably, the General Comment’s ‘availability, accessibility, acceptability and quality’ (AAAQ) framework,^46^ which was designed for health policy creation and implementation, and is a key feature of the right to health, receives explicit mention in the 2015 Act.

All of this needs to be seen in context: Colombia has a two-tier health system where people need to register in either a contributory or a subsidised regime to obtain comprehensive healthcare from private and public health providers. The system also has major structural issues, including funding, coverage in rural areas, and ensuring registration with and service provision-authorisation by insurers.^47^ Nonetheless, over the last 20 years, the personal health coverage of Colombian nationals has been significantly enhanced, access to benefits has increased for everyone, and essential medicines have generally been made more affordable.^48^

It is also noteworthy that over the last five years when deciding cases on the right to healthcare of the Venezuelan migrant population, the CCC has a pattern of references to international human rights law and practice, including by drawing on arguments put forward by amici curiae such as the human rights NGO De Justicia.^49^ Via these cases, the Court has declared that irregular migrants are entitled to: lifesaving treatment and urgent health care to prevent irreparable harm to life and health^50^; key vaccinations^51^; treatment for HIV/AIDS and cancer^52^; abortion^53^; prenatal check-ups and delivery care^54^; and comprehensive health care for all migrant children.^55^ It is an expansive caselaw; however, there are gaps—notably regarding the essential elements of a primary health care model which are core obligations that irregular migrants should enjoy.^56^

## IV. THE CASE STUDY

Now that we have sketched the TPSV and its backdrop, we want to dig deeper, testing both the regularisation scheme and our legal literacy hypothesis. Is Colombia’s response as good as it seems? Is it ‘a model of pragmatism and humanity’^57^ as suggested by the UN Refugee Agency, UNHCR, and the International Organization for Migration (IOM)? Is it a full-fledged human rights-based approach,^58^ harnessing international human rights law in ways that will reduce health inequalities between nationals and non-nationals? In what follows, we address these questions over five linked sections, beginning with the TPSV and then moving through other ways in which international human rights law is, and is not, having an impact.

### A. A model of pragmatism and humanity?

Regularised migration status is a key determinant of health: it enhances opportunities for socioeconomic wellbeing and integration, and is often—as in the Colombian case—a precondition for affiliation with the health system and access to healthcare.^59^ At the same time, as generations of legal realist and law-and-society scholars have demonstrated, ‘law in action’ can pull in different ways to ‘law in books’. Put differently, practice does not necessarily follow form or potential. So, what is the practice to date with the TPSV and the TPPs available thereunder?

A 2021 report by the Danish Council for Refugees suggests that a lack of awareness of health rights, procedures, and obligations among both service users and providers, as well as administrative barriers, remain significant hurdles to Venezuelan migrants’ right to health.^60^ At that time only 40 per cent of regular migrants were affiliated with the social security system.^61^ Recent figures show that of the 1.6 million Venezuelans who have received a TPP, 765,000 are registered with a health insurer.^62^ Thus the questions arising include: amidst both a formal regularisation scheme and a broader pro-immigrant political discourse, what reasons explains the missing 50 per cent?

It is clear that the scheme needs to wrangle the barriers that TPP-holding migrants face in making their healthcare rights real—from denial of health system registration by public and private insurers who are unfamiliar with the TPP,^63^ to xenophobia acting as direct barrier to accessing hospitals and clinics because security staff are turning away Venezuelan people.^64^ Extrapolating from this, and broadening out to other social determinants of health, banks, broadband companies, utility providers, chambers of commerce, and others need to recognise TPP-holders as individuals with formal legal status. Thus, in line with the AAAQ framework that is a key feature of the human right to health, the TPSV scheme needs to be accompanied by accessibility supports, including awareness campaigns designed to uphold information accessibility.^65^ At the same time, because it is much easier to apply for a TPP than to seek and obtain asylum in Colombia, and because the grant of a TPP requires Venezuelan asylum seekers to make a choice (renounce the TPP and continue with their asylum application or vice versa),^66^ close ongoing attention needs to be paid to whether the TPSV is routing individuals away from Colombia’s refugee recognition procedures in ways that interfere with the rights of refugees and asylum seekers.^67^

These gaps between TPSV form and practice, and the risk of inappropriate rerouting away from refugee recognition procedures, are not the only issues arising: the form of the scheme also raises at least three issues. First, although the government has promised the TPSV will be extended to other nationalities,^68^ currently only migrants of Venezuelan nationality are eligible. While states are sovereign in defining their migration policies, and the TPSV’s preferential treatment of Venezuelans may be a legitimate aim, it could also fall foul of international human rights law’s non-discrimination principle. The International Convention on the Elimination of All Forms of Racial Discrimination provides that states’ approaches to nationality, citizenship, and naturalisation should ‘not discriminate against any particular nationality’.^69^ The UN treaty body responsible for monitoring this Convention has emphasised that differential treatment on the grounds of nationality may be discrimination if immigration criteria are not applied ‘pursuant to a legitimate aim’ and are not ‘proportional to the achievement of this aim’.^70^ In Colombia, the CCC has previously determined cases involving alleged discrimination between national and nonnationals, and between regular and irregular migrants, regarding the enjoyment of human rights, holding that differential treatment will be permissible only where it is reasonable and proportionate. Thus, as things stand, litigation challenging the TPSV on the basis of the principle of non-discrimination cannot be ruled out.

Second, although the TPSV is designed for Venezuelan migrants, it does not extend to all such migrants: the eligibility criteria mean that the scheme creates a category of Venezuelan migrants for whom TPPs are unattainable (eg irregular migrants who have entered Colombia after the cut-off date of 31 January 2021). For this group, in the absence of a new regularisation programme, the Colombian government’s welcoming stance towards Venezuelan migrants amounts to nothing. Interviews with members of this group point to the particular burden of being excluded amidst ‘seemingly inclusive governmental contexts’: exclusion fosters ‘a sense of hopelessness about every acquiring legal residency’—‘most felt stuck in illegality’.^71^ Meanwhile, worryingly, those who are eligible and have received TPPs (or their predecessor SPPs), seem to have an undue sense of security—one that ‘anesthetised their urgency to activate resources and strategies to secure more enduring legality’.^72^ The third issue with the TPSV’s form points to a serious related problem: the scheme is built on executive orders, which means it offers no legal certainty—it can be terminated at any time by the current government or a future one. In addition, individual TPPs can be denied or withdrawn by Migración Colombia for reasons that include being deemed ‘inconvenient’.^73^ And because no provision has been made for administrative review of such decisions, the right to due process of law is in put in jeopardy.^74^

### B. Institutionalisation and accountability

One point emerging clearly from the previous section is that international human rights law does not simply establish rights and obligations: it calls for legal recognition of them. It also requires *institutionalisation* enabling the realisation of rights and it ‘presupposes and demands’ *accountability*.^75^ The reason for this is simple: even with formal legal recognition in legislation and caselaw, rights and obligations cannot count as rights and obligations without institutionalisation and accountability. These essential elements complete what the former UN Special Rapporteur on extreme poverty describes as a key framework for advancing socioeconomic rights—the recognition, institutionalisation, and accountability (RIA) framework:

(a) the need to accord legal recognition to rights; (b) the need for appropriate institutional arrangements to promote and facilitate realization of the right; and (c) the need for measures that promote governmental accountability.^76^

In Colombia, amidst the unprecedented wave of migration from Venezuela, the country’s highest court—the CCC—has been a central conduit to institutionalisation and accountability. As outlined in Section III, the Court’s caselaw—relying heavily on international human rights law—has sought to realise health rights and hold government accountable by highlighting gaps in irregular migrant rights’ protection.^77^ This caselaw also has more general significance for two reasons. First, across the world, many courts continue to resist engagement with economic and social rights, even in the face of constitutions that provide for justiciability. Second, the CCC counts among a growing number of courts worldwide that are being creative with remedies, seeking in particular to use ‘dialogical’ judgments (rather than judgments that seek to ‘command and control’ government) to catalyse deliberation and action by multiple actors.^78^ In so doing, the CCC and others are recognising that:

Judicalization is most successful when it intensifies political action, connecting litigation with social demands and participation, and in turn incentivizing democratic debates and actions by the executive and legislative branches.^79^

At the same time, and running contrary to the intense focus on judicial enforcement in right to health scholarship, institutionalisation and accountability via courts are not enough. The reasons for this include: courts deal only with the particular questions put before them, and access to justice through courts is not evenly distributed, even in a context like Colombia where *tutela* claims allow individuals to bring actions claiming violations of their fundamental rights (indeed overreliance on tutelas arguably undermines routes that allow collective claims and solutions).^80^ Furthermore, as Malcolm Langford and his co-authors emphasise, celebrating case law is one thing, ‘making it stick’^81^ is another—especially we would add in a field like health equity where implementation involves an intersectoral web of actors.^82^

Every one of our informants reported that ‘making it stick’ was an issue as regards the CCC’s extensive caselaw on urgent healthcare for irregular migrants. We were told that hospitals and health clinics normally offer only emergency care or stabilisation of vital signs: urgent care, which is defined both by the CCC and international human rights law as the care necessary to prevent critical harm to life, health and functionalities,^83^ is *not* routinely being provided to irregular migrants. Comprehensive access to maternal and child healthcare, which is also set out in CCC caselaw as well as national policy documents,^84^ is likewise far from being achieved in practice: several studies report health inequalities between Venezuelans and Colombians in these areas.^85^ And the position is reportedly worse again with respect to CCC caselaw establishing that HIV/AIDS care and treatment should be available to everyone.^86^

So what would be involved in ‘making it stick’? One key step, following the RIA framework, would be to secure statutory legal recognition of what falls within urgent care. This is important because it would curb the discretion of health operators in providing or refusing costly services to uninsured populations.^87^ Second, our interviews with key actors in the humanitarian field and local departments of health demonstrated either a lack of knowledge of these particular judgments, or unease with human rights law vocabulary, or the conviction that providing these health services would be too costly against the healthcare funding rules and impossible to realise without the creation of special healthcare routes.^88^ Institutionalisation and accountability therefore require more than judicial enforcement of rights; they require the design and implementation of special healthcare routes for irregular migrants seeking access to the level of care that the CCC has crystallised in its caselaw. This design and implementation process must involve both public institutions at different levels of government and private actors, as well as a focus on the material conditions of this disempowered group and ideally consultation with them.

### C. Legal empowerment

Interviews with staff at university legal clinics and the legal branches of NGOs revealed a ‘common sense’ concerning CCC judgments that ‘did not stick’, thus leaving gaps in urgent healthcare for irregular migrants: enjoying the right to urgent care required a legal or administrative action to be filed.^89^ Pro bono legal advisers reported making wide use of the *right of petition* procedure vis-à-vis public authorities or private entities responsible for negligent human rights implementation.^90^ One interviewee working near the capital city of Bogotá reported that, in her experience, ‘this remedy leads to successful replies and service provisions from the entity which refused the service, in 15 working days, in 80% of cases’.^91^ However, another interviewee cautioned that ‘this procedure may only work, in cases of denied urgent care, where health policies and practices are consistent and normally followed at local level, otherwise it is a waste of time’.^92^

If the right of petition mechanism is not a viable option in an individual case of unmet need for urgent healthcare, two other routes are available. The first is filing an *asylum application.* Colombia’s incorporation of the 1984 Cartagena Declaration on Refugees,^93^ a landmark in the development of the refugee protection regime in Latin America, means that these asylum applications can be grounded in ‘massive violation of human rights’ in Venezuela.^94^ If the application is declared admissible, the temporary document stating that the application is pending can be used to gain affiliation with the health system and, in principle, access to comprehensive care. Given that the average duration of asylum procedures is 2–3 years, this route can be the best option for migrants with complex and continuous health needs.

If an asylum application is not an option (eg because a person does not comply with asylum eligibility criteria such as having entered Colombia during the previous 60 days), filing a *tutela action* can be a good option to ensure urgent healthcare is accessible by individuals with irregular migrant status. The action allows any person, or their representative, to claim an irreparable violation of their fundamental rights by public and private entities before any ordinary judge, and the country’s highest court, the CCC, has jurisdiction as an appeal body in selected ‘tutela’ claims.^95^ A successful claim can injunct healthcare providers to offer a specific treatment and territorial health departments to pay the bill for such treatments. The latest available data shows that between 2018 and 2021, almost 4,000 such claims were filed by Venezuelan migrants before national judges, 80 per cent of which concerned health issues.^96^

There is a fourth route that can be used by legal clinics and NGOs to secure migrants’ health rights: the constitutionally established *actio popularis* for the protection of collective rights and interests.^97^ Its potential can be seen from a recent example of its use: in late 2021, the NGO Women’s Link filed an *actio popularis* claiming that migrant women in the region of Norte de Santander (bordering with Venezuela) were prevented from being informed about, and thus enjoying, sexual and reproductive rights. In response, the judge adopted an interim measure requiring the territorial health department and service providers to immediately coordinate and offer health services related to maternal care, termination of pregnancy, and sexual violence to all female migrants irrespective of their migration status.^98^

So if the rights of irregular migrants to urgent healthcare can be upheld via the above-listed legal routes, are we looking at an example of legal empowerment—of rights in action? On the one hand, it seems that the above-listed routes, particularly the tutela, offer ‘[t]ransparent, effective, and accessible accountability mechanisms’; as many have noted, mechanisms of this sort are ‘among the most important requirements of human rights, including the right to health’.^99^ On the other hand, some of these routes risk relentless objections claiming illegitimate expansion of judicial power—objections along the lines of ‘government by judges’.^100^ They also risk unintended consequences, potentially unbalancing health policy by foregrounding litigiousness and claims that are curative and largely private or individual. Further, although a focus on urgent care is essential, it is not enough: it represents a very constrained approach vis-à-vis health and social needs on the ground and the comprehensive care to which nationals and non-nationals are prima facie entitled according to human rights law.^101^

### D. Organisations as intermediaries and the rise of de facto rights

The just-described administrative and legal claims for the protection of the human rights of irregular migrants are often employed side by side with the provision of health services by humanitarian actors. The origin of this practice lies in intersectoral working designed to meet immediate health needs via humanitarian programmes, while a more durable legal rights-based solution is sought. The observation we conducted in migrant centres in the region Norte de Santander showed us that this intersectoral work of several humanitarian actors and territorial health agencies also affects other social determinants of health.

Thus, one question arising is: are the activities of Colombia’s many migrant-focused national and international humanitarian actors creating a *de facto* right to health for migrants with irregular or precarious status? Another question is: to what extent do *de facto* rights empower migrants and facilitate mobilisation for formal or *de jure* rights recognition?^102^ These are important questions given the ongoing resistance in many quarters to the idea of a right to health and the fact that, across human rights law scholarship, more attention has been paid to state-level actors and rights-holders than to the actors who mediate between legal and policy regimes and the experiences of individuals on the ground. Further, while human rights obligations ultimately rest with the state, seeking international aid and cooperation and liaising with charitable initiatives to realise the core health rights of people on the move—for example, via the provision of essential drugs, primary care, psychosocial support, food, water, and sanitation—is a strategy that complies with international human rights law.^103^

A structured humanitarian response emerged in Colombia from 2018 onwards, after the IOM and UNHCR announced their framework for an interagency response to the Venezuelan crisis. Known as the Refugee and Migrant Response Plan (RMRP), it was the first response of this kind in this part of the world. Humanitarian actors joined a Regional Coordination Platform for Venezuelan Refugees and Migrants (R4V), the Colombian branch of which (known as GIFMM, from its name in Spanish) includes 70 organisations.^104^ As was the case with previous versions, the top sectors and areas for intervention in the latest Response Plan of the R4V Platform constitute important determinants of health, including food, shelter, healthcare, sanitation, and protection from gender-based violence.^105^

The most recent data suggest that health interventions are amongst the most successful and well-funded, with quality primary healthcare services, mental health, and psychosocial support, as well as sexual and reproductive healthcare seen as the priorities.^106^ However, the data also suggest that GIFMM organisations are reaching just 15–40 per cent of people in need. This means that, regardless of the efforts of the many organisations in the field, meeting social and health needs, and significantly reducing inequalities in health determinants and human rights violations, for *all* migrant populations remains difficult to achieve in the short term.

As regards the health needs of irregular migrants, GIFMM organisations have adopted a three-fold strategy, coordinated overall with Colombia’s state/regional authorities.^107^ First, direct and contracted provision of primary and secondary health services, the former in a comprehensive way (including visits and treatment for common diseases, psychosocial support, nutrition services, vaccinations, sexual, and reproductive healthcare), the latter only sporadically when programme funding allows it. Second, both in and outside migrant attention centres, GIFMM organisations provide emergency accommodation, food and hygiene kits, transfer services, and legal and practical support regarding a number of intersectoral issues that constitute important determinants of health. Third, the organisations participate in referrals between organisations and to local authorities, in particular when protection issues arise.^108^

There is a learning opportunity here for making the right to health real for irregular migrants. On the one hand, as intermediaries between state actors and migrants, the GIFMM organisations and other independent actors are working in ways that forge *de facto* rights for irregular migrants. On the other hand, national and regional institutions are currently fully dependent on international and local non-state actors, and there is no trace of political willingness to progressively realise via state-funded programmes migrants’ rights as required under international human rights law.^109^ This is also a concern because the geographical coverage of humanitarian activities cannot embrace all territories, their actions targeting human rights are not legally accountable and remain dependent on funders’ priorities.

More broadly, the involvement of around 70 humanitarian organisations calls for close, ongoing attention to the way human rights—as a discourse, approach, or standards—frames programmes and interventions and informs the monitoring of results. The first impression, following interviews with some of these actors, is that human rights rhetoric or at best a mild human rights-based approach replace the use of clear human rights standards in their actions.^110^ This matters because projects targeting essential needs ‘might not be rights-promoting or even rights-protecting, and even when they are both, they may well end up promoting the special interests of a targeted group’ rather than the economic and social rights as human rights of all populations without discrimination on any ground, including nationality and legal status, and thus targeting equality in health.^111^ For instance, there is currently only one non-state organisation working on migrant disability needs and rights.^112^ Furthermore, while a significant share of current international funding and programmes seems to be focused on maternal healthcare, several informants noted there had been recent under-provision as regards other elements of the right to sexual and reproductive health (partly as a result of Trump-administration cuts in US financial and technical aid for sexual and reproductive services).^113^

Concluding this section, it is undeniable that humanitarian actors have contributed to the realisation of elements of the right to health as a *de facto* right, in particular around essential primary health care,^114^ yet their agenda, vocabulary, and priorities seem unlikely to contribute to mobilisation for *de jure* recognition of a broader right to health for irregular migrants.

### E. Participation

More positively, under the auspices of the IOM’s health and migration programme and in line with a national health policy priority,^115^ migrant people themselves, together with nationals living in their neighbourhoods, are developing a key role in health promotion activities and participating in realising critical elements of the right to health. They are doing this via community health networks: since 2018, 570 community leaders in 17 networks—though covering only a part of the state territory—have been engaged in activities around disease prevention, health education, and information, as well as identifying cases to be referred to the health system or humanitarian actors.

Realising the right to participation at community level, these leaders act as public health and human rights defenders in the field of health, thus facilitating the enjoyment of *de jure* and *de facto* rights to health for people living in their communities.^116^ The strength of the networks points to their potential as a first step towards the creation of fully formed community health committees with the potential to participate in health policymaking.^117^ Further, this capacity is being honed by training provided by several actors including Colombian universities. For example, the Universidad del Rosario, in Bogotá, is running a series of human rights recognition workshops during which community leaders engage in reflective and interactive exercises on their personal and collective experiences of health-related rights enjoyment and discrimination, as well as attending traditional training sessions on how to legally and administratively defend or ‘stand up’ for their rights.^118^

The potential of such initiatives is striking. This is community or grassroots leadership for human rights, supported through university-led human rights training. It is *not* human rights modelled on empowering people by empowering lawyers. It puts lawyers and other human rights professionals in a supporting role and, at its centre, are marginalised and vulnerable people seeking to build the power of their own community to name and transform health injustice.^119^

## V. CONCLUSION

It is 15 years after the WHO Commission on the Social Determinants on Health and almost 60 years after the International Covenant on Economic, Social and Cultural Rights. Each text acknowledges the obligation to address the social determinants of health; each text also gives a central place to equality and non-discrimination, and to the equally resonant value of human dignity. And yet social medicine/social epidemiology and international human rights law—the primary fields from which each text emerged—still engage only minimally with each other. As members of one of those fields, that struck us as a problem. This in turn led us to a hypothesis: all those concerned with health inequalities—the social injustice that is ‘killing people on a grand scale’^120^—need to ask more and better questions about international human rights law. We called this the legal literacy hypothesis and, to test it, we chose a challenging case study: Colombia’s response to a profound and protracted wave of migration from crisis-hit Venezuela.

The case study supports the hypothesis. Asking ‘the international law human rights question’ allowed us to flag problems with the form of the TPSV and with its operation to date in practice. It also allowed us to put the TPSV in context: by flowing out from this executive order, we were able to track how international human rights law is—and is not—influencing other key actors, including the CCC, legal clinics, humanitarian NGOs, and community health networks. We were in particular able to consider issues such as legal recognition, institutionalisation, and accountability.

The detail, as we explained earlier, is key. Legal literacy will be a non-starter if it collapses into generalisations about the impact of international human rights law. Legal literacy means acknowledging and accounting for *variables*. Thus, when the title of this article refers to ‘parsing’ human rights, we are calling not just for attention to law’s place within the broader human rights arena, but also to the background factors that influence law’s place. As the case study demonstrated, these factors can be expected to range well beyond formal texts such as constitutional provisions and executive orders. They must also include context such as the role of the judiciary, background legal structures (as eg, Colombia’s *tutela* and *actio popularis*), NGO mobilisation, involvement of rightsholders, and institutional arrangements for healthcare.

We are well aware that it is popular at present to dismiss human rights. And within human rights, we are concerned about a tendency to promote human rights-based approaches in ways that suppress law’s place. We do not want to see either trend deepening the low-level of engagement between practitioners of social medicine/social epidemiology and practitioners of international human rights law. It is well past time for a focus on international human rights law in the health inequalities literature, and correspondingly for more work on health inequalities, particularly the role of the social determinants of health and primary health care, in the literature on international human rights law. This can be achieved by looking at law as a determinant of health, or as a means of mitigating health inequalities.^121^ By way of a specific example, it can be by committing to health justice partnership—collaborations between legal and health professionals that facilitate the co-location of free legal support and healthcare services for vulnerable groups.^122^ Or, as we have argued, and overlapping with all of these, it can be taking up the challenge of legal literacy.

## Footnotes

1 World Health Organization (WHO), ‘Closing the Gap in a Generation: Health Equity through Action on the Social Determinants of Health: Final Report of the Commission on Social Determinants of Health’ (WHO 2008).

2 The small but growing literature includes L Forman, ‘International Human Rights Law and the Social Determinants of Health’ in S Benatar and G Brock (eds), *Global Health and Global Health Ethics* (2nd edn, CUP 2021).

3 For the most comprehensive statement of the right to health, see art 12 of the International Covenant on Economic, Social and Cultural Rights (ICESCR), opened for signature 19 December 1966, 993 UNTS 3. Non-binding but authoritative guidance on art 12 has been issued by the UN treaty body responsible for the Covenant: see Committee on Economic, Social and Cultural Rights (CESCR), ‘General Comment No. 14: The Right to the Highest Attainable Standard of Health (Art. 12 of the Covenant)’ (11 August 2000) UN Doc E/C.12/2000/4. Para 4 of this guidance explains that the right to health encompasses the underlying determinants of health, ‘such as food and nutrition, housing, access to safe and potable water and adequate sanitation, safe and healthy working conditions, and a healthy environment’.

4 For this criticism, see eg S Moyn, *Not Enough: Human Rights in an Unequal World* (Harvard UP 2019).

5 P Hunt, ‘Interpreting the International Right to Health in a Human Rights-Based Approach to Health’ (2016) 18(2) Health and Human Rights 109.

6 See eg UN Office of the High Commissioner for Human Rights (OHCHR) and Center for Economic and Social Rights, *Who Will be Accountable? Human Rights and the Post-2015 Development Agenda* (2013), delineating three dimensions of accountability: responsibility, answerability, and enforceability.

7 Constitución Política de Colombia [Political Constitution of Colombia] (Const.) (1991) art 93. See C Hübner Mendes,** **R Gargarella, and S Guidi (eds), *The Oxford Handbook of Constitutional Law in Latin America* (OUP 2022), ch 5 and the chapters in Part C.

8 M Baumgärtel, *Demanding Rights: Europe's Supranational Courts and the Dilemma of Migrant Vulnerability* (CUP 2019) 138 − 39.

9 C Dauvergne, *Making People Illegal: What Globalization Means for Migration and Law* (CUP 2009) 2.

10 Conducted by Stefano Angeleri.

11 Territorially, Colombia is divided into 32 departments and a capital district.

12 Profamilia, Red Somos, International Organization for Migration (IOM), Pan-American Health Organization, MedGlobal, Samaritan’s Purse, Humanity and Inclusion, Colombian Red Cross, Capellanía OFICA, Secretary of Health of the Department of Norte de Santander (2016 − 2019), Manager of ‘Health at the Border’ of the Department of Norte de Santander, Manitas Amarillas, Fundación RadaBer, Opción Legal, National Ombudsman on Human Mobility, UNICEF, Save the Children.

13 Previous discussions of this question include P Hunt, ‘Missed Opportunities: Human Rights and the Commission on Social Determinants of Health’ (2009) IUPHE Global Health Promotion (Supp. 1) 36; S Venkatapuram, R Bell, and M Marmot, ‘The Right to Sutures: Social Epidemiology, Human Rights, and Social Justice’ (2010) 12(2) Health and Human Rights Journal 3.

14 See eg UN Human Rights Council (UNHRC), ‘Report of the Special Rapporteur on Extreme Poverty and Human Rights, Philip Alston (Focus: Marginalization of Economic and Social Rights)’ (28 April 2016) UN Doc A/HRC/32/31 para 6, noting that many states continue to view socioeconomic rights as policy objectives and debates about these rights tend to ‘slide imperceptibly and almost naturally into broad discussions of development’.

15 Leading Lawrence Gostin, eg, to argue for ‘global health law *with governance*’ (emphasis added): see LO Gostin, *Global Health Law* (Harvard UP 2014).

16 See ICESCR (n 3) arts 2(2) and 3, read in conjunction with art 12, explicitly establish the immediate obligation to regulate the enjoyment of the health-related rights or provisions that exist at the domestic level in a non-discriminatory manner on a series of specified but non-exhaustive grounds, which have grown to include migrant status and nationality. See also GC14 (n 3) paras 30, 43(a).

17 See eg the work of the UN Special Rapporteur on extreme poverty and human rights, including UN General Assembly (UNGA), ‘Report of the Special Rapporteur on Extreme Poverty and Human Rights, Philip Alston (Focus: Digital Welfare States)’ (11 October 2019) UN Doc A/74/493; OHCHR, ‘Human Rights, Health, and Poverty Reduction Strategies’ (2008) Health & Human Rights Publication Series 5.

18 See eg UNGA, ‘Report of the Special Rapporteur on Extreme Poverty and Human Rights, Philip Alston (Focus: Privatization)’ (26 September 2018) UN Doc A/73/396; E Arenas Catalán, *The Human Right to Health Solidarity in the Era of Healthcare Commercialization* (Edward Elgar 2021).

19 A Sen, ‘Human Rights and the Limits of Law’ (2006) 27(6) Cardozo Law Review 2913, 2916.

20 See eg LE White and J Perelman (eds), *Stones of Hope: How African Activists Reclaim Human Rights to Challenge Global Poverty* (Stanford UP 2010); A Kapczynski, ‘The Right to Medicines in an Age of Neoliberalism’ (2019) 10 Humanity 79; G de Búrca, *Reframing Human Rights in a Turbulent Era* (OUP 2021).

21 SE Merry, *Human Rights and Gender Violence: Translating International Law into Local Justice* (University of Chicago Press 2005); B Simmons, *Mobilizing for Human Rights: International Law in Domestic Politics* (CUP 2009).

22 See eg P Hunt, S Bhalotra, and C Williams, ‘The Role and Impact of the Right to Health: Evidence from Brazil’s Family Health Program’ (2022) 44 Human Rights Quarterly 111, using interlinked administrative data for an entire population.

23 G de Búrca, ‘Legal Mobilization for Human Rights: An Introduction’ in G de Búrca (ed), *Legal Mobilization for Human Rights* (OUP 2022) 1−11, 11. See further de Búrca (n 20).

24 Interagency Coordination Platform for Refugees and Migrants from Venezuela (R4V), Key Figures (last update 10 January 2022) <https://www.r4v.info/en> accessed 13 December 2022.

25 ‘ENCOVI—Encuestas sobre condiciones de vida’ [Survey on Living Conditions] is a project conducted by three Venezuelan universities (Universidad Simón Bolívar, Universidad Central de Venezuela, and Universidad Católica Andrés Bello), which has monitored living conditions in Venezuela since 2014 <https://www.proyectoencovi.com/encovi-2021> accessed 13 December 2022.

26 Amnesty International, ‘Venezuela: Hunger, Punishment and Fear, the Formula for Repression used by Authorities under Nicolás Maduro’, News (20 February 2019) <https://www.amnesty.org/en/latest/news/2019/02/venezuela-hunger-punishment-and-fear-the-formula-for-repression-used-by-authorities-under-nicolas-maduro/> accessed 13 December 2022; Inter‐American Commission on Human Rights (IACmHR), ‘Preliminary Observations and Recommendations of the Country Visit to Venezuela on Human Rights Implementation’, press release no 106/2020 (8 May 2020) <http://www.oas.org/es/cidh/prensa/comunicados/2020/106.asp> accessed 13 December 2022.

27 WHO for Americas, ‘PAHO’s Response to Maintaining and Effective Technical Cooperation: Agenda in Venezuela and Neighbouring Member States’, 56th Directing Council/70th Session of the WHO Regional Committee (5 September 2018).

28 Migración Colombia, ‘Mas de dos milliones 447 mil venezolanos …’ [More than 2 million 447 thousand Venezuelans ...] Press Release (19 July 2022) <https://www.migracioncolombia.gov.co/noticias/mas-de-dos-millones-477-mil-venezolanos-se-encuentran-radicados-en-colombia-y-de-ellos-el-96-busca-hacer-parte-del-estatuto-temporal-de-proteccion-visibles> accessed 13 December 2022.

29 See eg A Selee and J Bolter, ‘An Uneven Welcome: Latin American and Caribbean Responses to Venezuelan and Nicaraguan Migration’, report, Migration Policy Institute, February 2020, 7 − 11 <https://www.migrationpolicy.org/research/latam-caribbean-responses-venezuelan-nicaraguan-migration> accessed 13 December 2022.

30 R4V, Key Figures (n 24).

31 See Decree no 216/2021 (1 March 2021); Resolution no 971/2021 (28 April 2021).

32 Decree no 216/2021, arts 4, 8, and 12; Resolution no 971/2021, arts 2, 5, 6, 15, and 16.

33 Resolution no 6045/2017, art 21.

34 M Marmot and others, ‘Fair Society, Healthy Lives: The Marmot Review’ (2010) 104.

35 Resolution no 971/21, arts 7, 8, and Title IV and V.

36 Decree no 216/2021; Resolution no 971/2021.

37 Decree no 216/2021, Preamble.

38 ibid.

39 Resolution no 971/2021, Preamble, art 14.

40 Const, art 93; CCC, Judgment C-750/2008.

41 CCC, Judgment C-067/2003.

42 H Alviar García, ‘Distribution of Resources led by Courts’ in H Alviar García, K Klare, and LA Williams (eds), *Social and Economic Rights in Theory and Practice: Critical Inquiries* (Routledge 2015) 67; Mendes,** **Gargarella, and Guidi (n 7) Part C.

43 CCC, Judgment T-760/2008. See further A Arrieta-Gómez, ‘Realizing the Fundamental Right to Health through Litigation: The Colombian Case’ (2018) 20(1) Health and Human Rights Journal 133.

44 Congress, Health Act (Ley Estatutaria en Salud) no 1751/2015 (16 February 2015) arts 1 and 6, codifying a right to health based on 14 principles: universality, pro homine, equity, continuity, promptness, age-sensitivity, progressive enhancement, free choice, sustainability, solidarity, efficiency, interculturalism, indigenous-sensitivity, and protection of minorities.

45 CCC, Judgment T-760/2008.

46 GC14 (n 3) para 12.

47 EM Herrera, CD Pulecio and MSR Barrero, ‘Propuesta para mejorar la eficiencia del Sistema General de Seguridad Social en Salud en Colombia’ [Proposals to Enhance the Efficiency of the General Social Security System in Colombia] (2021) 12 Revista CIES Escolme 287; E Lamprea and J García, ‘Closing the Gap Between Formal and Material Health Care Coverage in Colombia’ (2016) 18(2) Health and Human Rights Journal 45, 49.

48 Alviar García (n 42) 87–94.

49 Eg CCC, Judgments T-210/2018 and T-246/2020.

50 Eg CCC Judgment T-210/2018, para 34.

51 Eg CCC, Judgment T-025/2019, para iii. As regards COVID-19 vaccinations, via Resolution no 1255/2021 (20 August 2021), the Ministry of Health extended the vaccination campaign to all residents, irrespective of their migration status. However, it was October 2021 before the Minister announced that all irregular migrants were eligible <https://www.minsalud.gov.co/Paginas/El-pais-debe-avanzar-en-la-vacunacion-de-poblacion-migrante.aspx> accessed 13 December 2022. As of late October 2021, only 5.3 per cent of all Venezuelans in Colombia had received at least one dose according to the R4V, RMRP—2022 Regional Refugee and Migrant Response Plan (December 2021) 107.

52 Eg CCC, Judgments T-025/2019; T-197/2019; T-403/2019.

53 Eg CCC, Judgment C-055/2022.

54 Eg CCC, Judgments SU-677/2017, paras 56–57; T-074/2019, para 8.

55 Eg CCC, Judgment T-106/2022.

56 CESCR, GC14 (n 3) para 43; S Angeleri, ‘Access to Health Care for Venezuelan Irregular Migrants in Colombia: Between Constitutional Adjudication and Human Rights Law’ (2021) (early online date) International Journal of Human Rights.

57 UNHCR/IOM, ‘UNHCR and IOM Welcome Colombia’s Decision to Regularize Venezuelan Refugees and Migrants’ (8 February 2021) <http://www.unchr.org/news/press/2021/2/60214cf74/unchr-iom-welcome-colombias-decision-regularize-venezuelan-refugees-migrants.html> accessed 13 December 2022.

58 A human rights-based approach aims to empower rights-holders and strengthen the capacity of duty-bearers. It is underpinned by the five PANEL principles, wherein L stands for ‘legality’ (i.e., acting in accordance with the legal rights set out in domestic and international laws): see <https://ennhri.org/about-nhris/human-rights-based-approach/> accessed 13 December 2022.

59 IOM, ‘Migration: A Social Determinant of the Health of Migrants’, Background paper (IOM Migration Health Department 2006). For Colombia, see AM Murillo-Pedrozo and others, ‘A Qualitative Study of the Health Perceptions in the Venezuelan Immigrant Population in Medellín (Colombia) and its Conditioning Factors’ (2021) International Journal of Environmental Research and Public Health 5.

60 Consejo Danés para Refugiados [Danish Council for Refugees], Análisis de la Garantía de Derechos a la Educación, Salud e Inclusión Laboral de la Población Migrante de Venezuela en Colombia [Analysis of the Enjoyment of the Rights to Education, Health and Economic Integration of Venezuelan Migrants in Colombia] (March 2021) 25. See more generally also CCC, Judgment T-178/2019, paras 24, 28, 41.

61 Webinar Presentation of Mr Espinosa, director of Migración Colombia, at the Irish Centre for Human Rights/Embassy of Colombia to Dublin webinar ‘Colombia’s Approach to the Venezuelan Migrant Crisis’ (26 May 2021).

62 Ministry of Health—National Observatory of Migration and Health, ‘Statistics on Migrant Affiliation’ (31 August 2022) <https://www.sispro.gov.co/observatorios/onmigracionysalud/Paginas/Migrantes-afiliados-a-Salud.aspx> accessed 13 December 2022.

63 Informal interview with IOM staff during participant observation activities in Norte de Santander (25 February 2022), as confirmed by L Ramirez, speaker at the virtual event Colombia +20/El Espectador ‘How is the sexual and reproductive health of migrant population?’ (3 March 2022).

64 Informal interviews with staff at IOM and Jesuit Refugee Service during participant observation activities in Norte de Santander (16−25 February 2022). Xenophobia against Venezuelan people grew dramatically during the COVID-19 pandemic: Universidad Externado de Colombia, Barómetro de la Xenofobia [Xenophobia Barometer] trimestral report Colombia (April–June 2021) <https://issuu.com/elderechoanoobedecer/docs/informe_segundo_trimestre_2021_-_bx_oficial_> accessed 13 December 2022.

65 See GC14 (n 3), para 12(b).

66 Decree no 216/2021, art 17.

67 See S Angeleri and MT Palacios Sanabria, ‘A Rights-based Assessment of the Temporary Protection Statute for Venezuelans in Colombia’ (2002) 47 Yale Journal of International Law Online 81, 91−93.

68 L Suesca ‘Estatuto de Protección se extenderá a otras nacionalidades’ [The protection statute will extend to other nationalities] Caracol Radio (2 August 2022) <https://caracol.com.co/radio/2022/08/02/politica/1659453925_027502.html> accessed 13 December 2022.

69 International Convention on the Elimination of All Forms of Racial Discrimination (CERD), opened for signature 21 December 1965, 660 UNTS 211, art 1(3).

70 CERD Committee, ‘General Recommendation No 30: Discrimination against Non-Citizens’, Sixty-fifth session (2005) para 4.

71 D Del Real, ‘Seemingly Inclusive Liminal Legality: The Fragility and Illegality Production of Colombia’s Legalization Programmes for Venezuelan Migrants’ (2022) 48 Journal of Ethnic and Migration Studies 3580, 3590, 3595.

72 ibid.

73 Decree no 216/2021, arts 2 and 15(3).

74 Angeleri and Palacios Sanabria (n 67) 89.

75 Alston (n 14) para 8.

76 ibid para 21. See also AE Yamin, ‘On Principle and Persuasion: Examining Philip Alston’s Contribution to Economic and Social Rights through the Lens of Health’ in N Bhuta and others (eds), *The Struggle for Human Rights* (OUP 2021).

77 See text at nn 50−55 above.

78 See CCC, Judgment T-760/2008, adopting structural litigation guidelines that directed government to tackle major issues in the country’s healthcare system. For discussion, see eg KG Young and J Lemaitre, ‘The Comparative Fortunes of the Right to Health: Two Tales of Justiciability in Colombia and South Africa’ (2013) 26 Harvard Human Rights Journal 179.

79 Yamin (n 76) 186.

80 See e.g. the *actio popularis* discussed below at text accompanying (nn 96–97). For details on the Colombian tutela, see Young and Lemaitre (n 78) 184–86.

81 M Langford, C Rodriguez-Garavito, and J Rossi (eds), *Social Rights Judgements and the Politics of Compliance: Making it Stick* (CUP 2017).

82 UN Secretary-General, Secretary-General’s Address to the Commission on Human Rights (7 April 2005).

83 For this approach to urgent care, see CCC, Judgment T-210/18; International Convention on the Protection of the Rights of All Migrant Workers and Members of Their Families (ICMW), opened for signature 18 December 1990, 2220 UNTS 3, art 28; UN Human Rights Committee, *Toussaint v Canada* (24 July 2018) UN Doc CCPR/C/123/D/2348/2014.

84 Ministerio de Salud de la República de Colombia/Ministry of Health, ‘Plan de Respuesta del Sector Salud al Fenómeno Migratorio’ [Response Plan to Migration for the Health Sector] (Bogotá 2018) 49.

85 LJ Bonilla-Tinoco, M Aguirre-Lemus and JA Fernández-Niño, ‘Venezuelan Migrant Population in Colombia: Health Indicators in the Context of the Sustainable Development Goals’ (2020) 9 F1000Research 684; CC Guarnizo-Herreño and GL Wehby, ‘Health of Infants Born to Venezuelan Refugees in Colombia’ (2021) 23 Journal of Immigrant & Minority Health 222.

86 Danish Refugee Council, ‘Acceso a servicios de salud de personas refugiadas y migrantes que viven con VIH en Bogotá [Access to Health Services for Refugees and Migrants living with HIV in Bogota] (March 2022); CCC, Judgments T-025/2019; T-197/2019; T-403/2019.

87 JM Amaya-Castro, C Moreno and G Pelacani, ‘La gestión de la migración en Colombia: Propuesta de dialogo para una política pública con enfoque de derechos en educación y salud’ [Migration Management in Colombia: Proposing Public Policies with a Rights-based Approach to Education and Health], Informe [Report] (Centro de Estudios en Migración 2019) <https://derecho.uniandes.edu.co/sites/default/files/Informe-cem.pdf> accessed 13 December 2022.

88 Interviews with humanitarian NGOs in the department of Norte de Santander and ombuds offices in Bogotá (May 2022).

89 Interviews with legal branches of an NGO in Cúcuta - Norte de Santander and a community-based NGO in Bogotá (April 2022).

90 Const, art 22; Congress Act no 1755 of 2015 (30 June 2015).

91 Informal interview during participant observation with an international NGO working in Soacha in Cundinamarca (April 2022).

92 Semi-structured interviews with a community organisation in Bogotá (May 2022).

93 Cartagena Declaration on Refugees, opened for signature 22 November 1984, para 3.3. Beyond the definition in the 1951 UN Refugee Convention and the 1967 Protocol, the Declaration extends who should be considered a refugee to: ‘persons who have fled their country because their lives, safety or freedom have been threatened by generalised violence, foreign aggression, internal conflict, massive violation of human rights or other circumstances which have seriously disturbed public order.’

94 Ministry of Foreign Affairs, Decree no 2840 of 6 December 2013, art 1.

95 Const, arts 81, 241(9).

96 Consejo de Redacción, ‘La tutela en Colombia, un salvavidas para la salud de los venezolanos’ [‘tutela’ in Colombia, lifesaving claims for the health of venezuelanas] (2021) citing the CCC <https://consejoderedaccion.org/Especiales/salud-en-el-exilio/tutela-Colombia-salvavida-salud-venezolanos.html> accessed 13 December 2022.

97 Const, art 88; Congress Act no 472 of 1998 and no 1425 of 2010.

98 Women’s Link, ‘Entidades públicas de salud de Norte de Santander deben tomar acciones inmediatas para garantizar la salud sexual y reproductiva de las mujeres de la región’ [Public bodies in Norte de Santander must take immediate actions to ensure sexual and reproductive health for women] (press release, 10 November 2021) <https://www.womenslinkworldwide.org/informate/sala-de-prensa/entidades-publicas-de-salud-de-norte-de-santander-deben-tomar-acciones-inmediatas-para-garantizar-la-salud-sexual-y-reproductiva-de-las-mujeres-de-la-region> accessed 13 December 2022.

99 Hunt, Bhalotra, and Williams (n 22) 115.

100 Alston (n 14) para 40 outlines the following accountability options over and above courts: ‘(a) sharing information with the media; (b) using community or peer pressure; (c) collecting and publishing data; (d) complaining to an authoritative body or person; and (e) evaluating and reporting.’

101 See eg the core and immediate obligations outlined in GC14 (n 3), and focus on ‘essential primary health care’ ever since CESCR, ‘General Comment no 3: The Nature of States Parties Obligations’ (14 December 1990) UN Doc E/1991/23 para 10. See also CCC, Judgment T-210/2018, para 47 holding that state authorities are under the duty to progressively move towards the equalisation of healthcare for *all migrants* and nationals. Four years later, Colombia’s legal framework lacks a clear timeframe and plan for achieving the promised health rights enlargement for all migrants, regardless of their legal status.

102 See relatedly CA Heimer and AW Tolman, ‘Between the Constitution and the Clinic: Formal and De Facto Rights to Healthcare’ (2021) 55 Law & Society Review 563.

103 ICESCR (n 3) art 2; CG14 (n 3) para 38.

104 R4V/GIFMM membership is available from the Platform’s website <https://www.r4v.info/es/colombia> accessed 13 December 2022.

105 R4V, RMRP 2022 (n 51) 54.

106 ibid; R4V, RMNA 2022 Refugee and Migrant Needs Analysis (October 2022) 35.

107 Operating in line with cooperation agreements and authoritative guidelines for humanitarian organisations: see IOM and Colombian Government, ‘Country Strategy of the IOM Colombia—period 2021–2024’, pt 39, at <https://colombia.iom.int/sites/g/files/tmzbdl1011/files/documents/Estrategia%20Pa%C3%ADs%20OIM%20Colombia%202021-2024FK-2.pdf> accessed 13 December 2022; United Nations Population Fund, ‘Country programme document for Colombia—period 2021-2024’ (2020), DP/FPA/CPD/COL/7, pt 20−23; Sphere Association, *The Sphere Handbook: Humanitarian Charter and Minimum Standards in Humanitarian Response* (4th edn, 2018); <https://www.spherestandards.org/handbook> accessed 13 December 2022.

108 Informal interviews with staff at the IOM, Jesuit Refugee Service, Health Institute of the Department of North of Santander, UNICEF, Action against Hunger, UNFPA, during direct and participant observation activities in Norte of Santander (16−25 February 2022).

109 Interviews with humanitarian actors and public servants in Cúcuta in Norte de Santander (May 2022).

110 Interviews with humanitarian actors in Bogotá (May−June 2022).

111 Alston (n 14) para 6. See also M Broberg and H-O Sano, ‘Strengths and Weaknesses in a Human Rights-Based Approach to International Development: An Analysis of a Rights-based Approach to Development Assistance Based on Practical Experiences’ (2018) 22 International Journal of Human Rights 664; F Bustreo and CFJ Doebbler, ‘The Rights Based Approach to Health’ in LO Gostin and BM Meier (eds), *Foundations of Global Health & Human Rights* (OUP 2020) 89 − 110.

112 Interview with a humanitarian organisation in Bogotá (May 2022).

113 Interview with humanitarian actors in Bogotá and Cúcuta.

114 GC3 (n 101) para 10; GC14 (n 3) para 43.

115 Ministry of Health, Directorate of Prevention and Promotion, ‘Guidelines for the creation and strengthening of health community networks in the framework of public health and collective interventions’ (December 2015) <https://www.minsalud.gov.co/sites/rid/Lists/BibliotecaDigital/RIDE/VS/PP/ENT/orientaciones-redes-sociales-comunitarias-pic.pdf> accessed 13 December 2022.

116 IOM Colombia, ‘17 community networks act for health and wellbeing promotion in 24 territories of Colombia’ press release (28 July 2021) <https://colombia.iom.int/es/news/17-redes-comunitarias-actuan-en-pro-de-la-salud-y-el-bienestar-en-24-territorios-de-colombia> accessed 13 December 2022.

117 M Mulumba and others, ‘Using Health Committees to Promote Community Participation as a Social Determinant of the Right to Health: Lessons from Uganda and South Africa’ (2018) 20(2) Health and Human Rights Journal 11.

118 One of the authors, Stefano Angeleri, is one of the coordinators of this pilot project of the IOM and Universidad del Rosario.

119 See M Satterwhaite, ‘Critical Legal Empowerment’ in de Búrca (n 23) describing what seem to be similar moves—including community paralegals—in a range of places around the world.

120 WHO (n 1).

121 See eg R Magnusson, ‘Advancing the Right to Health: The Vital Role of Law’ (WHO 2017); A Phelan and R Magnusson, ‘Update and Summary Guide to the Report “Advancing the Right to Health”’ (WHO 2018) <https://apps.who.int/iris/handle/10665/275522> accessed 13 December 2022; Lancet-O’Neill Commission on Global Health and Law, ‘The Legal Determinants of Health: Harnessing the Power of Law for Global Health and Sustainable Development’ (30 April 2019) <https://www.thelancet.com/commissions/legal-determinants-of-health> accessed 13 December 2022.

122 H Genn, ‘When Law is Good for Your Health: Mitigating the Social Determinants of Health Through Access to Justice’ *Current Legal Problems* (OUP 2019).

